# Rapid Reversal of Human Intestinal Ischemia-Reperfusion Induced Damage by Shedding of Injured Enterocytes and Reepithelialisation

**DOI:** 10.1371/journal.pone.0003428

**Published:** 2008-10-17

**Authors:** Joep P. M. Derikx, Robert A. Matthijsen, Adriaan P. de Bruïne, Annemarie A. van Bijnen, Erik Heineman, Ronald M. van Dam, Cornelis H. C. Dejong, Wim A. Buurman

**Affiliations:** 1 Department of Surgery, School for Nutrition & Metabolism (NUTRIM), Maastricht University Medical Centre+, Maastricht, the Netherlands; 2 Department of Pathology, Cardiovascular Research Institute (CARIM), Maastricht University Medical Centre+, Maastricht, the Netherlands; Centre de Recherche Public-Santé, Luxembourg

## Abstract

**Background:**

Intestinal ischemia-reperfusion (IR) is a phenomenon related to physiological conditions (e.g. exercise, stress) and to pathophysiological events (e.g. acute mesenteric ischemia, aortic surgery). Although intestinal IR has been studied extensively in animals, results remain inconclusive and data on human intestinal IR are scarce. Therefore, an experimental harmless model for human intestinal IR was developed, enabling us to clarify the sequelae of human intestinal IR for the first time.

**Methods and Findings:**

In 30 patients undergoing pancreatico-duodenectomy we took advantage of the fact that in this procedure a variable length of jejunum is removed. Isolated jejunum (5 cm) was subjected to 30 minutes ischemia followed by reperfusion. Intestinal Fatty Acid Binding Protein (I-FABP) arteriovenous concentration differences across the bowel segment were measured before and after ischemia to assess epithelial cell damage. Tissue sections were collected after ischemia and at 25, 60 and 120 minutes reperfusion and stained with H&E, and for I-FABP and the apoptosis marker M30. Bonferroni's test was used to compare I-FABP differences. Mean (SEM) arteriovenous concentration gradients of I-FABP across the jejunum revealed rapidly developing epithelial cell damage. I-FABP release significantly increased from 290 (46) pg/ml before ischemia towards 3,997 (554) pg/ml immediately after ischemia (p<0.001) and declined gradually to 1,143 (237) pg/ml within 1 hour reperfusion (p<0.001). Directly after ischemia the intestinal epithelial lining was microscopically normal, while subepithelial spaces appeared at the villus tip. However, after 25 minutes reperfusion, enterocyte M30 immunostaining was observed at the villus tip accompanied by shedding of mature enterocytes into the lumen and loss of I-FABP staining. Interestingly, within 60 minutes reperfusion the epithelial barrier resealed, while debris of apoptotic, shedded epithelial cells was observed in the lumen. At the same time, M30 immunoreactivity was absent in intact epithelial lining.

**Conclusions:**

This is the first human study to clarify intestinal IR induced cell damage and repair and its direct consequences. It reveals a unique, endogenous clearing mechanism for injured enterocytes: rapid detachment of damaged apoptotic enterocytes into the lumen. This process is followed by repair of the epithelial continuity within an hour, resulting in a normal epithelial lining.

## Introduction

Intestinal ischemia-reperfusion (IR) injury is an important (patho)physiological mechanism, potentially leading to a compromised mucosal barrier in numerous situations [1], [2]. Intestinal hypoperfusion occurs during exercise both in healthy relatively untrained volunteers and in highly trained athletes, which stresses the abdominal vasodilator reserve by diverting blood flow from the splanchnic circulation to the exercise muscles [3]–[5]. This substantial reduction in blood flow is sometimes associated with symptoms of abdominal hypoperfusion, which are almost always reversible without intensive therapy [3]–[5]. Stress may also lead to symptomatic intestinal ischemia via increased catecholamine release, resulting in severe splanchnic vasospasm [6]. Furthermore, intestinal IR may be looked at as a common pathway in several pathologies, where temporary reduction in flow may result from inherent vascular disease (thrombosis, shock, embolism, vasculitis) or from inflammatory diseases affecting perfusion. An example of inherent vascular disease is acute mesenteric ischemia (AMI), which carries a high morbidity and mortality rate, both increased by frequent delays in diagnosis. Such diagnostic delays are mainly caused by non-specific signs and symptoms and limited diagnostic accuracy of the laboratory and radiological tests currently in use. Arterial and venous thromboembolisms are the main causes of AMI [1], [2]. More global hypoperfusion may result from shock. Along these lines, intestinal IR is considered to be a crucial phenomenon involved in the onset of necrotizing enterocolitis, in the pathogenesis of small intestinal transplantation and rejection, and in the potential development of postoperative or posttraumatic complications, such as systemic inflammatory response syndrome, sepsis and multiple organ failure [2], [7], [8]. Here, inflammation and collateral damage may play a role.

Generally, IR injury starts with affecting cell metabolism, ultimately resulting in cell death (apoptosis as well as necrosis) [9]–[11]. Upon reperfusion, ischemia primed cells are prone to generate toxic reactive oxygen metabolites and release of constitutive cellular proteins upon loss of cell membrane integrity, which can act as danger signals and activate the immune system [9]–[15]. In the resulting inflammatory response, local expression of adhesion molecules, proinflammatory mediators and activation of neutrophils as well as the complement system are central mediators of IR induced tissue injury [10], [11], [13]–[16]. This proinflammatory state increases tissue vulnerability to further injury, resulting in the amplification and derailment of the inflammatory response, although it is meant to be a beneficial reaction to clear damaged cells [11], [14], [16].

The pathophysiology of intestinal IR has hitherto mainly been studied in animal models (rodents, cats, dogs and pigs) by transient clamping of the superior mesenteric artery [17]–[19]. First event after intestinal IR in all these animals is the appearance of subepithelial spaces at the villus tips, immediately followed by loss of the highly susceptible mature enterocytes [17], [18]. Depending on the duration of ischemia, this sloughing may continue toward the crypt, causing denuded villi [17], [18]. In line with general IR-induced cell damage, also in intestinal IR, apoptosis is the major mode of cell death in the destruction of epithelial cells [20]. Moreover, the disruption of the interaction between epithelial cells and extracellular matrix (‘anoikis’) may play an important role in the onset of apoptosis in detached enterocytes [20]. Some studies report that after longer periods of reperfusion, migration of epithelial cells to seal the exposed basement membrane occurs, which closes the loss of epithelial continuity [19], [21]. Further studies show that reperfusion leads to inflammation, resulting in transmural infarction [19], [22]–[24]. This inflammatory response is comparable to the response of other organs to IR induced cell damage, including infiltration of neutrophils and activation of complement [22], [24].

The histological consequences of human gut IR are known from patients operated upon for different gastrointestinal disorders after obvious signs of clinical shock or from post-mortem studies [25]. Microscopic examination of gut segments of these patients all showed characteristic mucosal lesions, ranging from epithelial lifting at the villus tip to denuded villi with disintegrated and hemorrhagic lamina propria [25]. The histological findings concerned end-stages of diseases and were identical to the previously described ultimate phases of intestinal mucosal IR-induced damage in animals.

This study was directed at revealing the sequelae of intestinal IR in man in the course of time, using a newly developed human experimental, controlled model of 30 minutes intestinal ischemia, followed by variable periods of reperfusion.

## Methods

### Patients and experimental, surgical procedure

Thirty patients (12F∶18M) with a median age of 68.5 years (range: 35–79 years) undergoing pancreatic surgery were studied. Twenty-eight of these patients underwent a standard pylorus-preserving or classical pancreatico-duodenectomy (Whipple's procedure) for benign or malignant disease, whilst two patients underwent a Frey procedure for chronic pancreatitis. During pancreatico-duodenectomy a variable length of jejunum is usually resected in continuity with the specimen. Similarly, in creating a Roux-limb for the pancreatico-jejunostomy in Frey's procedure, it is often necessary to resect a small segment of jejunum that is less well perfused. We took advantage of this, which enabled us to study IR induced cell damage in a harmless human jejunal IR model. To that purpose, the most distal part of the jejunum to be resected with the Whipple specimen, or the most proximal part of the Roux-limb to be used as a pancreatico-jejunostomy in the Frey procedure, was used to isolate a 6 cm segment of jejunum. The isolated jejunum (6 cm) was subjected to 30 minutes ischemia followed by a median reperfusion time of 128 minutes (range: 0–210 minutes) according to the following experimental protocol.

In short, the first part of surgery (opening of the abdominal wall, installation of a large self-retaining retractor and exploration for metastases and localization of the major anatomical structures and tumour) was according to standard procedures. From this moment the 6 cm part of jejunum, which was going to be studied was identified and care was taken that the vasculature of the studied jejunum consisted of 1 central mesenteric arteriole and venule. This was achieved by dissection, clamping and cutting of all collateral mesenterial vessels to the studied jejunum using Ultracision Harmonic Ace (Johnson&Johnson, cat. no. ACE23P, Amersfoort, the Netherlands). Thereafter, the segment of jejunum was further isolated by transsection at both ends with a linear cutting stapler (GIA 6038S, Covidien, Zaltbommel, the Netherlands). The isolated jejunum was then subjected to 30 minutes ischemia with 2 atraumatic vascular clamps, which are placed over the mesentery (Bulldog, Aesculap, cat. no. BH013R, Tuttlingen, Germany). The isolated ischemic jejunum was subsequently placed in the abdominal cavity to guarantee warm ischemia. The surgical procedure proceeded as planned. After half an hour of ischemia, one third (2 cm) of the isolated ischemic jejunum was resected using linear cutting stapler to study early phenomena during ischemia. Reperfusion was initiated by removal of the clamps, and the isolated jejunum was inspected for restoration of blood flow. A further segment of isolated jejunum (2 cm) was resected similarly after 25 minutes of reperfusion to study early phenomena during reperfusion. The last part of studied jejunum was resected from 60 minutes after reperfusion onwards to investigate late phenomena during reperfusion. At the time the last isolated reperfused jejunum was obtained for the study, also 2 cm of jejunum, which had not been isolated and remained untreated during the surgery, was resected using a linear cutting stapler. This tissue was used as internal control tissue: it was from the same patient and experienced similar surgical handling as the isolated jejunum, while it was not subjected to IR. Then, our study-protocol was ended and surgery continued as anticipated.

### Histological assessment

The tissue specimens obtained at four different time points during the study (after half an hour of ischemia, after 25 minutes of reperfusion, from 60 minutes reperfusion onwards and internal control) were immediately immersed in 3.7%–4.0% formaldehyde fixation (Unifix, Klinipath, Duiven, the Netherlands) and incubated overnight at room temperature. Next, the formalin fixed samples were embedded in paraffin and 4 µm sections were cut. Sections were stained with haematoxylin and eosin (H&E) using standard histological techniques.

During the conduct of the experiments, it was noted that in every jejunal sample we collected at the time of maximal reperfusion, a milky substance appeared from the lumen. In order to study the content of this substance, we collected and fixed it according to the AgarCyto cell block procedure [26]. Shortly, the collected cells were fixed in Unifix (Klinipath) for 24 hours. Fixed cells were centrifuged for 5 minutes at 1200 rpm. The pellet was carefully resuspended in 1 ml 2% liquid agarose at 65°C (LE, analytical grade; Promega, Madison, WI) and transferred into a 1.5-ml Eppendorf tube. The tube was centrifuged for 5 minutes at 1000 rpm to concentrate the cells in the agar. The agar–cell pellet was solidified at 4°C for at least 1 hour. The agar cone was carefully taken out of the reaction tube with pointed forceps and divided in two halves in the sagittal plane of the cone. The two agar pieces were embedded in paraffin under standard conditions for surgical biopsies. This paraffin embedded agar–cell pellet is called AgarCyto [26]. From the AgarCyto, 4 µm sections were cut. For cytomorphologic examination, AgarCyto sections were stained with H&E.

### Immunohistochemistry

Paraffin sections were dewaxed in xylene and rehydrated in graded ethanol to distilled water. Endogenous peroxidase activity was blocked using 0.3% hydrogen peroxide in methanol for 12 minutes. Sections undergoing heat-mediated antigen retrieval were placed in a cooker filled with 10 mM citrate buffer (pH 6.0) for 10 minutes for staining with collagen IV. After blocking the non-specific antibody binding using 5% BSA, the sections were incubated with specific primary antibody at room temperature for 45 minutes. The following primary antibodies were used: mouse anti-cleaved cytokeratin 18 antibody, clone M30 (Catalogue no. 10700; Peviva, Bromma, Sweden); mouse anti-Collagen IV, clone CIV 22 (Catalogue no. M0785; DAKO, Glostrup, Denmark); mouse anti-smooth muscle actin (SMA), clone 1A4 (Catalogue no. M0851; DAKO); and chicken anti-intestinal-fatty acid binding protein (I-FABP, kindly provided by Hycult Biotechnology (HBT; Uden, the Netherlands)). After washing, an appropriate biotin-conjugated secondary antibody was used. Binding of the primary antibody was demonstrated by the streptavidin-biotin system (DAKO) and visualized by applying 3-amino-9-ethylcarbazole (AEC; Sigma, St. Louis, MO). For I-FABP, peroxidase-conjugated rabbit anti-chicken IgY (Catalogue no. 303-035-003; Jackson ImmunoResearch Europe, Suffolk, UK) was used as secondary antibody and AEC was used as chromogen. For staining of collagen IV, labelled polymer detection agent (PowerVision Poly-HRP-anti-Mouse/Rabbit/Rat IgG (Immunologic; Duiven, The Netherlands)) was applied followed by AEC. Nuclei were counterstained with haematoxylin. The stained slides were photographed by a Nikon eclipse E800 microscope with a Nikon digital camera DXM1200F. No significant staining was detected in slides incubated with control rabbit serum and/or mouse IgG instead of the primary antibody indicating the absence of significant background staining.

### Immunofluorescence

Cryostat sections (4 µm) were cut and stained for Zona Occludens-1 (ZO-1). Briefly, slides were dried, fixed in 4% paraformaldehyde for 15 minutes and non-specific antibody binding was blocked using 10% normal goat serum. Next, slides were stained with anti ZO-1 (catalogue no. 61-7300; Zymed Laboratories Inc., San Francisco, CA). Slides were incubated with a Texas Red labelled secondary antibody (Jackson, West-Grove, PA). Subsequently, slides were mounted using glycerol-PBS with 1, 4-diazabicyclo (2, 2, 2) octane (DABCO) and 4, 6-diamidinol (2)-phenylindole (DAPI), and viewed with an immunofluorescence microscope.

### Blood sampling and I-FABP measurement

Arterial blood was sampled preoperatively, before the isolated jejunum was subjected to ischemia, immediately upon reperfusion and every half hour during reperfusion until the end of the study protocol. Simultaneous with the second (i.e. before ischemia) and every next arterial blood sample, blood was drawn from the venule draining the isolated jejunal segment by direct puncture to assess concentration gradients across the isolated jejunal segment. All blood samples were directly transferred to pre-chilled EDTA vacuum tubes (BD vacutainer, Becton Dickinson Diagnostics, Aalst, Belgium) and kept on ice. At the end of the study all blood samples were centrifuged at 4000 rpm, 4°C for 15 minutes to obtain plasma. Plasma was kept on ice and immediately stored in aliquots at −80°C until analysis.

Intestinal Fatty Acid Binding Protein (I-FABP) concentrations were measured in plasma by means of the commercially available enzyme-linked immunosorbent assay (ELISA) used according to manufacture guidelines and kindly provided by HBT. I-FABP arteriovenous concentration differences were calculated (mesenteric venule minus radial artery) before and after ischemia to assess epithelial cell damage.

### Ethics

The study was approved by the Medical Ethics Committee of the Maastricht University Medical Centre + and all individuals gave written informed consent.

### Statistics

Statistical analysis was performed using Prism 4.0 for Windows (GraphPad Software Inc. San Diego, CA). I-FABP arteriovenous concentration gradients were presented as mean±standard error (SEM). Normality of all data obtained was verified by Kolmogorov-Smirnov test. Bonferroni's multiple comparison test was used (after significant repeated measures ANOVA) to compare I-FABP arteriovenous concentration differences in time. A p-value below 0.05 was considered to be statistically significant.

## Results

### Early phenomena during ischemia

**Figure 1:**
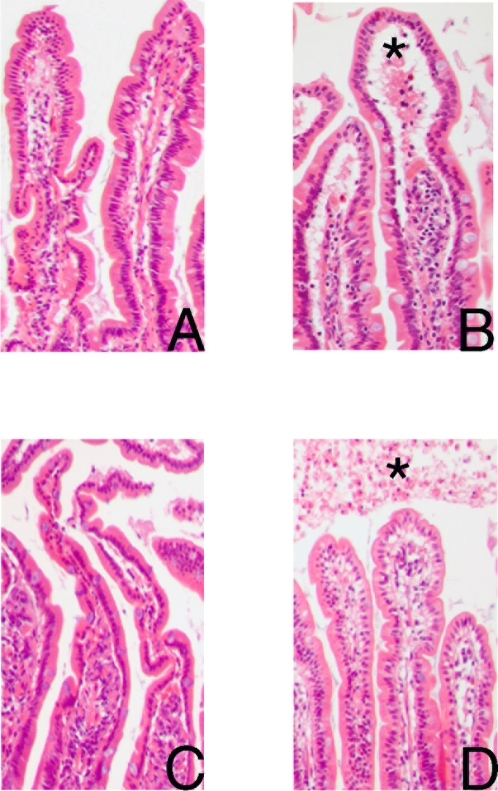
Histological analysis of H&E-stained jejunal sections (100×) shows a normal epithelial lining in untreated tissue (A), and upon 30 minutes of ischemia (B). However, subepithelial spaces (*) appear after 30 minutes ischemia (B). After 25 minutes reperfusion shedding of mature enterocytes into the lumen is found (C). Within 60 minutes of reperfusion the epithelial lining is resealed, while debris of apoptotic, shedded enterocytes (*) are found in the lumen (D).

**Figure 2:**
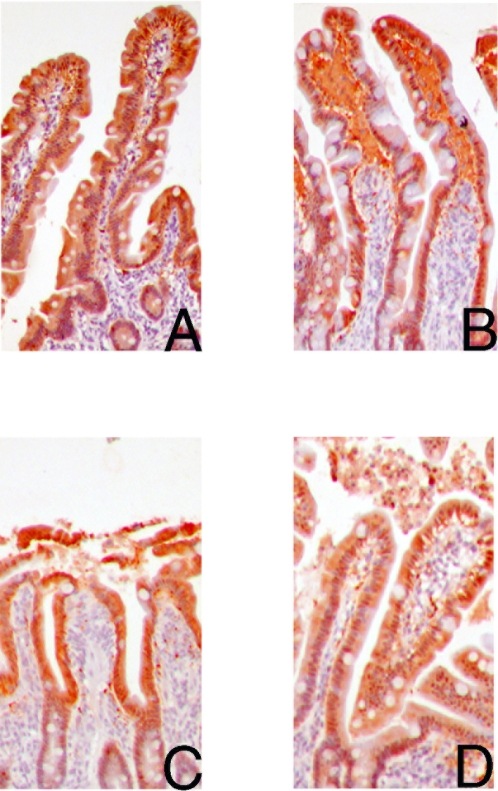
Immunolocalization of I-FABP in red (3-amino-9-ethylcarbazole, AEC) (100×) in the control jejunum not subjected to ischemia-reperfusion (A) shows an abundant cytosolic presence of I-FABP in the epithelial cells of the upper half of the villus. Upon 30 minutes ischemia (B), cytosolic I-FABP staining is decreased in mature enterocytes with abundant staining in the subepithelial spaces. A decreased cytosolic staining is still observed after 25 minutes reperfusion (C). Within 60 minutes reperfusion, I-FABP cytosolic positive cells are part of the resealed epithelial barrier (D), while shedded I-FABP containing enterocytes are found in the debris in the lumen.

At the end of the ischemic period of 30 minutes, H&E sections of the jejunum showed microscopically normal epithelial lining (Figure 1A, B). However, subepithelial spaces appeared at the villus tip. To study the epithelial cells in more detail, immunohistochemical staining of I-FABP was performed, a small protein present in the cytoplasma of differentiated enterocytes [27]. Further, we stained ZO-1, a 225 kDa membrane bound protein, binding the transmembrane tight junction proteins occludin and claudins and linking them to cytoskeletal actin [28]. Intensive immunostaining for I-FABP was observed in the control jejunal epithelium, mainly in the cytoplasm of the mature enterocytes and goblet cells in the upper half of the villi, whereas cells in the crypts were not stained, as previously observed in experiments by our group (unpublished results) (Figure 2A). A decreased staining of I-FABP was observed in jejunal mature epithelial cells, after 30 minutes ischemia, while intense staining was found in the subepithelial spaces (Figure 2B), indicating early leakage of I-FABP from the cytoplasm of intestinal epithelial cells into the subepithelial space. ZO-1 was detected at the apical pole of the epithelial cells in both the control jejunum and the jejunum after an ischemic period of 30 minutes (data not shown).

**Figure 3:**
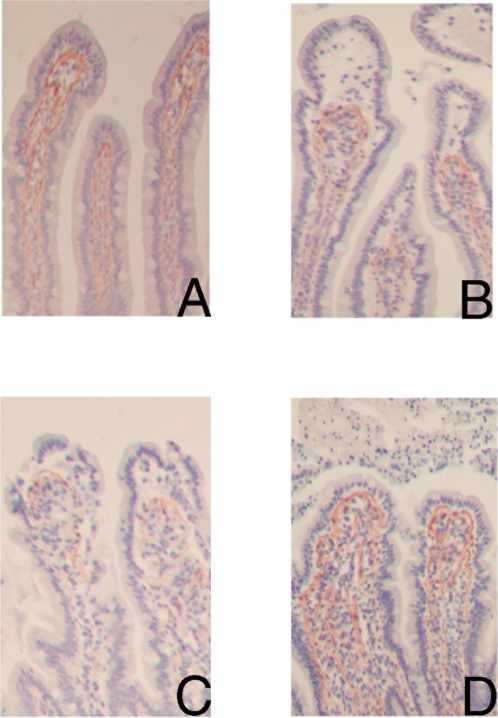
Characterization of the basement membrane with collagen IV staining in red (AEC) shows collagen IV positive cells directly beneath the epithelial layer in control jejunum (100×). (A). Upon 30 minutes of ischemia, a clear retraction is found of the collagen IV positive cells from the basal pole of the epithelial cells at the tip of the villus, causing subepithelial spaces (B). After 25 minutes reperfusion, the retracted basement membrane is still observed (C). Within 60 minutes reperfusion, the collagen IV positive basement membrane is again attached to the epithelial lining (D).

To clarify the development of the subepithelial spaces, the basement membrane, demarcating the lamina propria from the epithelial cells, and the underlying network of myofibroblasts within the villus lamina propria were studied, by staining collagen IV and smooth muscle actin (SMA), respectively [29], [30]. A clear retraction of the basement membrane was found from the basal pole of the epithelial cells at the tip of the villi (Figure 3A, B). In line with this, shorter myofibroblasts were observed in a denser lamina propria (data not shown).

### Early phenomena during reperfusion

**Figure 4:**
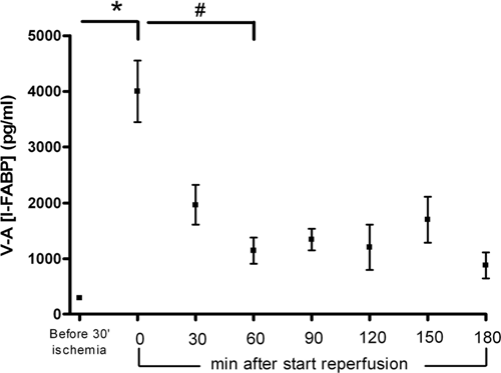
Arteriovenous concentration differences of I-FABP (mesenteric venule minus radial artery), a marker for acute enterocyte damage, across the isolated jejunal segment show rapid significant release of I-FABP after ischemia, while concentration differences of I-FABP gradually reduce following reperfusion. * p<0.001, # p<0.001.

To investigate loss of enterocyte membrane integrity, arteriovenous concentration differences of I-FABP across the studied jejunum were measured before ischemia and from reperfusion onwards. All individuals showed a basal release of mean (SEM) I-FABP of 290 (46) pg/ml from the isolated jejunal segment before ischemia, potentially reflecting the physiologic turnover of enterocytes (Figure 4). Interestingly, I-FABP release significantly increased towards 3,997 (554) pg/ml immediately after ischemia and upon reperfusion (p<0.001), suggesting rapid epithelial cell damage following 30 minutes ischemia. A gradual, significant decline to 1,143 (237) pg/ml of I-FABP release occurred within 1 hour reperfusion (p<0.001, immediately upon ischemia vs. 1 hour reperfusion).

**Figure 5:**
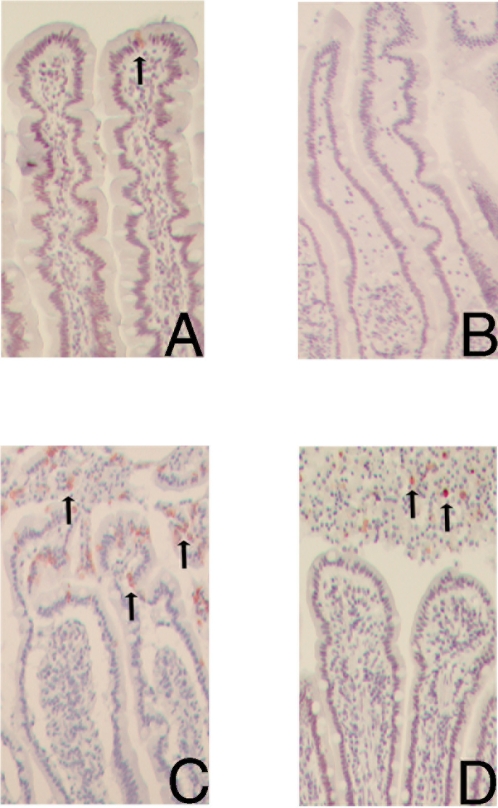
Apoptosis, assessed by M30 staining in red (AEC). An M30 positive cell (arrow) is observed at the top of the villus in normal jejunum (100×) (A). Upon 30 minutes ischemia, no M30 positive staining is found (B), while after 25 minutes reperfusion intense M30 immunostaining is observed at the villus tip near the shedding of mature epithelial cells into the lumen (arrows) (C). At 60 minutes reperfusion M30 immunoreactivity is no longer detectable in the intact, resealed epithelial barrier, while debris of M30 positive, shedded epithelial cells is observed in the lumen (D).

After 25 minutes of reperfusion, structural damage to the mucosa became apparent, consisting of detachment of epithelial cells, particularly in the apical regions of the villi, leading to shedding of (sheets of) mature epithelial cells into the lumen (Figure 1C). Intestinal villus epithelial cells, which had lost the contact with the underlying villous stroma, underwent a phenomenon called ‘anoikis’, which refers to detachment-induced apoptosis [31]. Therefore, apoptosis was visualized in the jejunal sections using the apoptosis marker M30, which detects the Asp396 caspase cleavage site in cytokeratin-18 [32]. Intense M30 immunostaining was observed after 25 minutes of reperfusion at the villus tips, at the site where extensive desquamation of the epithelial cells from the villus tips into the intestinal lumen occurred, which was not detected in the earlier phase (Figure 5A–C). In the shortly reperfused jejunum a substantial loss of ZO-1 expression was shown in the sheets of shedded cells, which was accompanied by a decreased cytoplasmatic immunoreactivity of I-FABP.

### Late phenomena during reperfusion

**Figure 6:**
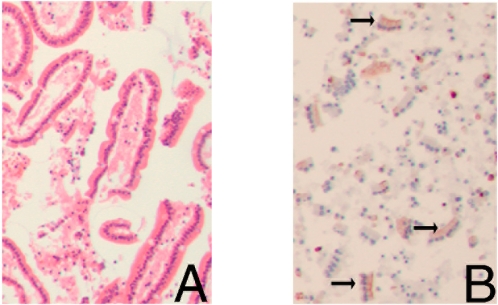
Histological analysis of H&E stained AgarCyto-fixed debris, appearing from the lumen of the jejunum after 30 minutes ischemia and more than 60 minutes ischemia, shows sheets of enterocytes (100×). (A), containing many M30 positive cells (arrows) in red (AEC) (100×) (B).

As mentioned in the Materials and Methods section, a notable finding in all jejunal samples collected after 30 minutes ischemia and more than 60 minutes reperfusion was the presence of a milky substance that appeared from the lumen. Histological analysis of this debris revealed sheets of enterocytes with numerous apoptotic cells, assessed by H&E and M30 staining (Figure 6A, B).

The H&E staining of the jejunum, which underwent 30 minutes ischemia and more than 60 minutes reperfusion, confirmed the debris of detached epithelial cells in the lumen (Figure 1D). Interestingly, within 60 minutes reperfusion the epithelial layer resealed and full confluent coverage of the villus tip by enterocytes was observed again, indicating a recovered epithelial barrier (Figure 1D). The cells coating the lamina propria were immunopositive for I-FABP and ZO-1, indicating that the epithelial continuity contained viable enterocytes with intact tight junctions (Figure 2D). Immunostaining with collagen IV showed that the basement membrane was again attached to the epithelial lining (Figure 3D). To investigate the status of these epithelial cells, M30 immunostaining was performed to assess apoptosis. M30 immunoreactivity was absent in the newly formed intact epithelium, while the debris of shedded epithelial cells in the lumen contained apoptotic cells (Figure 5D).

## Discussion

This study for the first time provides insight in the sequence of events taking place during intestinal ischemia-reperfusion (IR) in man. Intestinal IR is often encountered in clinical practice, not only in patients presenting with abdominal complaints, but also during e.g. exercise, major surgery and trauma [1]–[4], [8]. Patients undergoing pancreatico-duodenectomy enabled us to create the first human experimental model allowing detailed studies of intestinal IR induced cell damage, because in this procedure a variable length of healthy jejunum is removed as part of the standard operative procedure. This controlled model completely equals the interruption of blood flow to the small intestine, which often occurs during repair of a (thoraco-) abdominal aneurysm of the aorta, cardiopulmonary bypass, and intestinal transplantation.

Our study reveals that three phenomena can be distinguished following 30 minutes of human intestinal ischemia. Firstly, during the ischemic period the epithelial integrity remained seemingly intact, while subepithelial spaces appeared by detachment of the basement membrane from the enterocytes without any indication for the onset of apoptosis. The presence of I-FABP staining in the subepithelial spaces strongly indicates loss of membrane integrity of the intestinal epithelial cells at the site of formation of subepithelial spaces. These data are in line with the rapid increase of arteriovenous concentration differences of I-FABP immediately upon ischemia. Secondly, within 30 minutes reperfusion, desquamation of the intestinal epithelium at the tip of the villi was observed into the lumen, accompanied by apoptosis of mature epithelial cells. Thirdly, within 60 minutes reperfusion, a fully intact epithelial lining was found, indicating rapid restoration, whereas the apoptotic enterocytes had been shedded in the lumen. This reaction of the intestinal villi upon ischemia reperfusion, which collectively is called restitution, was also found in previous in vitro and in vivo animal studies after various insults, as recently reviewed by Blikslager et al [19]. However, the consequences of intestinal IR in animal studies vary largely and seem to be dependent on the animal model, the animal strain, preoperative care (e.g. starvation/fasting, premedication) and duration of ischemia or hypoperfusion [17]–[19].

Intestinal epithelial restitution is hypothesized to be directed at repairing the epithelial defect, which originates from the detachment of damaged enterocytes. The appearance of subepithelial spaces during ischemia is the actual start of the regeneration and aimed at reducing the effective size of the injured area. The development of subepithelial spaces after splanchnic hypoperfusion in animal studies was initially interpreted as an accumulation of cytoplasmic fluid from ischemic damaged cells [17]. Later, evidence was provided by an in vitro model of chemically-induced injury to guinea pig ileal epithelium that the subepithelial space resulted from the retraction of the basement membrane by contraction of myofibroblasts [29]. This retraction leads to a smaller and denser lamina propria accompanied by some seepage of fluid from the lamina propria cells into the newly formed subepithelial space. This is in line with our observations that the subepithelial spaces developing after 30 minutes of ischemia were associated with a dense lamina propria and a liquid-containing space between the basement membrane and enterocytes. Immediately after the start of the reperfusion, the I-FABP staining of the subepithelial space strongly indicates a loss of enterocyte membrane integrity, resulting in leakage of I-FABP into the subepithelial space. In line, we observed immediately upon ischemia strongly elevated arteriovenous concentration gradients of I-FABP, reflecting the membrane integrity loss of the epithelial cells of the villus. Therefore, we hypothesize that the enterocyte cell membrane must have lost its integrity during ischemia.

The mature enterocytes at the tip of the villus are the cells that are most susceptible to IR [33]. This has classically been explained by their constant state of hypoxia due to the counter current exchange mechanism of oxygen in the villus microvasculature, wherein oxygen from arterial blood entering the villus diffuses across to neighbouring venules travelling from the tip down toward the base of the villus [33]. As a result of this phenomenon, a steep oxygen gradient is present in the intestinal villus with substantially lower oxygen tensions in the villus tip than at the crypt [33]. In addition, desquamation occurs of the damaged mature enterocytes from the tip of the villi, resulting in shedding of sheets of epithelial cells into the lumen. Apoptosis is the major form of cell death observed after the onset of reperfusion and at the villus tip. Apoptosis of detaching enterocytes after intestinal IR is potentially caused by loss of contact between the epithelial cells and extracellular matrix, and between neighbouring epithelial cells (‘anoikis’) [20], [31], [34]. Extracellular matrix molecules provide a constant survival signal to epithelial cells via β1-integrins and cadherins as well as their adhesion-mediated signalling pathways including focal adhesion kinase (p125fak), phosphatidylinositol 3′-kinase (PI3-K)/Akt and mitogen-activated protein kinase (MAPK; p38) [34]–[37]. However, senescent enterocytes gradually loosen cell anchorage by changes of integrin expression, cadherin binding and its signalling pathways, making them prone to be shedded from the villus tip at the end of their lifespan, a mechanism crucial for maintenance of homeostasis in this epithelium [35]–[37]. Moreover, acyl-coenzyme A synthetase 5, which is solely expressed at the villus tip, is able to sensitize epithelial cells to apoptosis specifically triggered by the death ligand TRAIL [38]. Therefore, mature enterocytes are in an anti-adhesive and pro-apoptotic state when they reach the villus tip, culminating in the physiologic shedding [39]. This may contribute to the rapid onset of apoptosis that we observed in the mature and damaged enterocytes after the ischemic period, upon reperfusion.

The ultimate reparative response to the lost epithelial continuity was observed within one hour of reperfusion, consisting of a complete resealing of the epithelial defect overlying the basement membrane by viable, ZO-1 and FABP containing, epithelial cells. The presence of tight junctions and endogenous cytosolic proteins in the repaired epithelial cells of the villus tip indicates that the epithelial lining is rapidly restored, which is of importance to reduce the opportunity for luminal microbiota and their products to translocate. The question remains whether this also leads to a reestablishment of the full barrier function, which will be assessed in future using physiological measurements by tracer or electrophysiological studies. Previous studies reported that the migration of epithelial cells involves alteration of enterocyte shape and phenotype, including extension of the plasma membrane in the direction of cell migration and assembly of new focal contacts at the leading edge [19], [40], [41]. Epithelial migration during restitution is enhanced by luminal contents and a variety of systemic and local factors, including trefoil peptides, polyamines and transforming growth factor β [19], [42].

The clearing mechanism of IR-induced injured cells is unique to the intestine. In other organs or tissues, such as heart, brain, kidney, liver and skeletal muscle, IR-induced damaged and death cells remain in situ, since they cannot be removed from the organs or tissues and cleared from the body. In such organs, an inflammatory response is elicited amongst others to clear away the injured and death cells after IR [10], [13]–[15], [43]. Subject of further studies is the investigation of the inflammatory response in our controlled, experimental model of human IR, which enabled us to clarify a gut-specific protective mechanism for IR-induced damaged cells. Our results might explain why the gut can tolerate 30 minutes of ischemia in events as exercise, trauma, repair of a (thoraco-) abdominal aneurysm of the aorta, and cardiopulmonary bypass.
